# Book review: Helping people with eating disorders: a clinical guide to assessment and treatment (Second Ed)

**DOI:** 10.1186/s40337-016-0101-7

**Published:** 2016-04-04

**Authors:** Josie Geller

**Affiliations:** Research, Eating Disorders Program, St. Paul’s Hospital, Vancouver, Canada; Department of Psychiatry, University of British Columbia, Vancouver, Canada

**Keywords:** Book review, Eating disorders, Recovery, Guidebook

## Abstract

ISBN: 978-1-118-60668-1, 304 pages, June 2014, Wiley-Blackwell

Replete with insight and passion, the second edition of “Helping People with Eating Disorders,” reads like an in-depth consultation from a wise and trusted colleague. Anyone who knows Bob Palmer will appreciate that his voice is clearly heard throughout this book. The in-depth review of evidence, commentary and expert opinion can only arise from years of clinical work and experience in the field.

The book has as its stated aim to give clinicians an overview of current evidence-based treatment in the eating disorders. It is clear, intelligent, and authoritative while cognizant of the limits to our knowledge and understanding. With its thoughtful perspective on existing evidence, this book addresses the over-certainty that can exist in the field, offering a wise and balanced perspective. The use of clinical case studies and personal commentaries gives richness to this informative read. This book will serve as an invaluable resource for clinicians.

Helping People with Eating Disorders is divided into two sections. Within a framework of ‘what’ comes before ‘why,’ in the first section Dr. Palmer describes key features and characteristics of the diagnostic criteria for eating disorders (ED). Specifically, topics include who suffers and asks for help, the causes of EDs, thinking about EDs, and what is involved in recovery. Using cases that illustrate the diagnostic categories, Dr. Palmer details the difficulties patients encounter. The second section focuses on the “Why” and how to help. It includes sections on assessment, pointers for Bulimia Nervosa, Anorexia Nervosa, unusual disorders, what can go wrong, and organizing services. Alive with clinical examples and personal commentaries found in the notes at the end of each chapter, this book serves as a guiding hand through the literature.

While the book is an easy read, “Helping People with Eating Disorders” was written for those who have “chosen- or found themselves chosen – to offer help to those suffering from clinical eating disorders.” That is to say, it assumes a level of familiarity with both mental health and medical terminology. It identifies limitations in our current understanding and offers suggestions for future directions. For example, Dr. Palmer writes that over the years, our field has become focused on biological mechanisms and determinants of eating disorders. While acknowledging the use of fascinating new technology, he explores its clinical utility and suggests that the most meaningful goal for the field would be to integrate biology, psychology, technology and clinical observation.

Professor Palmer’s signature warmth, human and personal approach make this book an invaluable resource to all eating disorder clinicians.

